# Source Contributions of PM_2.5_ in the Severe Haze Episode in Hebei Cities

**DOI:** 10.1155/2015/480542

**Published:** 2015-11-04

**Authors:** Zhe Wei, Litao Wang, Simeng Ma, Fenfen Zhang, Jing Yang

**Affiliations:** Department of Environmental Engineering, School of City Construction, Hebei University of Engineering, Handan, Hebei 056038, China

## Abstract

Beijing-Tianjin-Hebei area is one of the most polluted areas in China. This paper used the Fifth-Generation Penn State/NCAR Mesoscale Model (MM5) and Model-3/Community Multiscale Air Quality (CMAQ) modeling system to quantify the source contribution to PM_2.5_ in Hebei cities in order to obtain an in-depth understanding haze process in January and February 2013, using the Multiresolution Emission Inventory for China (MEIC). The result showed that PM_2.5_ were mainly originated from the southern Hebei (SHB) with the fractions of 70.8% and 66.4% to Shijiazhuang, 70.6% and 63.9% to Xingtai, and 68.5% and 63.0% to Handan in January and February 2013, respectively. The northern Hebei (NHB) contributed 69.8% and 70.7% to Zhangjiakou, 68.7% and 66.2% to Chengde, and 57.7% and 59.6% to Qinhuangdao in January and February. In Cangzhou, Hengshui, and Langfang, regional joint policy making should be implemented due to the pollution of multiple sources. In Baoding and Tangshan, industrial emissions contributed 38.1% and 41.9% of PM_2.5_ to Baoding and 39.8% and 45.8% to Tangshan in January and February, respectively. Industrial and domestic emissions should be controlled in Tangshan and Baoding, especially for industrial emissions of NHB.

## 1. Introduction

An extreme regional haze episode with extensive influence area and high particulate matters (PM) concentration has occurred in centre-eastern China during January 2013, which has attracted wide attentions and concerns over the world. This haze event is the most serious pollution event since 1961 [1], and the average concentration of PM_10_ has reached the largest value since 2001 [2]. Large amounts of studies have discussed this haze episode in terms of the characteristics, the formation processing, source contributions, chemical compositions of particulate matters, and so forth [1–7]. The stagnant meteorological conditions and typical terrain resulted in accumulation of particulate matters, leading to severe and lasting haze episode [8]. Hebei was one of the most polluted provinces in China in January of 2013, which is related to the huge emissions in Hebei and the surrounding regions. According to the Asian INTEX-B emission inventory [9], the PM_2.5_ emission from Hebei, Henan, Shandong, and Shanxi accounted for 28% of national total emission in 2006. The percentages for SO_2_, NO_*X*_, CO, VOC, BC, and OC were 28%, 25%, 28%, 24%, 30%, and 24%, respectively [10]. In 2011, 45.5% of the steel in the world was produced in China, 24.0% of which was produced in Hebei. Hebei's cement production accounted for 6.9% of the national total amount [11]. China's coke production accounted for more than 60% of the world, 14.5% of which was produced in Hebei [12]. All of the data gives a clue why Hebei province has the most severe air pollution all over China. The daily average concentration has even attained 300 *μ*g m^−3^ in Shijiazhuang [8] in January 12, 2013, which has notably exceeded Chinese Grade II standard (75 *μ*g m^−3^) [13]. The coexistence of multipollutants, the superposition of multiple sources, the poor meteorological conditions, increase of secondary components, and the typical terrain have prompted the severe formation of this haze event [3, 14].

Along with economic development and environmental deterioration, the government looks forward to quantify the source contributions of PM_2.5_. Several cities have released the detailed source apportionment results of PM_2.5_, as shown in Table 1. Due to different geography locations and energy structure among those cities, the source contributions of sectors to PM_2.5_ present different characteristics. Briefly, local emission is the major contributor. Wang et al. [15, 16] applied the Mesoscale Modeling System Generation 5 (MM5) and the Models-3/Community Multiscale Air Quality (CMAQ) evaluate the source contributions to PM_2.5_ in the three southern Hebei cities, for example, Shijiazhuang, Xingtai, Handan, from January to February 2013 and found that the local emission (approximately 65%) was of great importance to PM_2.5_ in Shijiazhuang and Xingtai. In Hebei province, the source contributions of PM_2.5_ have not been finished and published except for Shijiazhuang mentioned in Table 1. Few studies have reported the source contributions of PM_2.5_ in other cities of Hebei, but this work is important. Therefore, this paper applied MM5-CMAQ model to quantify the source contributions of PM_2.5_, which was necessary for policy making and air pollution control.

## 2. Model Configurations and Inputs

### 2.1. Model Domain and Episode

**Figure 1:**
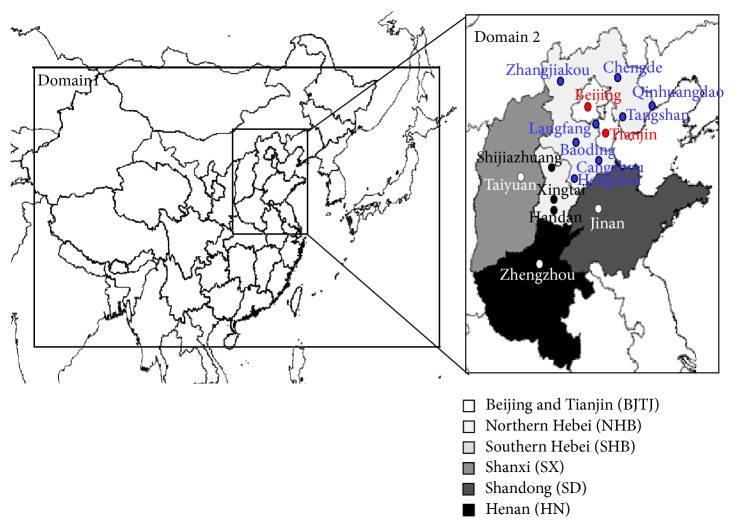
The model domain (Hebei province is divided into the southern Hebei (SHB, which includes Shijiazhuang, Xingtai, and Handan) and northern Hebei (NHB, which includes Zhangjiakou, Chengde, Qinhuangdao, Tangshan, Langfang, Baoding, Cangzhou, and Hengshui).

An offline MM5-CMAQ model is preformed over two nested domains: as shown in Figure 1, East Asia with a grid resolution of 36 × 36 km (Domain 1) and an area in northeastern China encompassing Beijing, Tianjin, and the four provinces, Hebei, Henan, Shandong, and Shanxi at a 12 × 12 km (Domain 2). The origin of domain 1 is 34°N and 110°E. The simulated results of domain 1 are employed for providing the boundary and initial conditions so as to running domain 2. January and February 2013 are selected to calculate the source contributions of PM_2.5_, because the monthly average concentration of PM_2.5_ in January 2013 is the highest since 2001, and the number of days of haze is maximum since 1961. The simulations of February are used to compare the source contribution of PM_2.5_ with the severe haze period of January. Additionally, a spin-up period of 5 days (27–31 December 2012) is applied to minimize the influence of the initial conditions.

### 2.2. Model Configurations and Data Input

Using MM5 model (version 3.7) combined with four-dimensional data assimilation (FDDA) to produce meteorological field for CMAQ model. The input data of MM5 and terrain and land use data are drawn from US Geological Survey Database (ftp://ftp.ucar.edu/mesouser/MM5V3/TERRAIN_DATA/), which provides initial and boundary field for CMAQ. First guess field with 1°  × 1° resolution, 6-hour interval, and the initial conditions are extracted from the US Geological Survey Database. The observation data use the National Center for Environmental Prediction (NCEP) Final (FNL) Operational Global Analysis datasets. The major physics options used in the MM5 simulations include the Kain-Fritsch 2 cumulus scheme, the high resolution Blackadar PBL scheme, and the mixed phase explicit moisture scheme for cloud microphysics, the cloud atmospheric radiation scheme for both long-wave and short-wave radiation, and the force/restore surface scheme. The MM5 output files are postprocessed by the Meteorology-Chemistry Interface Processor (MCIP) version 3.6 on an hourly basis [17]. The vertical distribution includes 23 sigma stories, and sigma story of the closest land is equal to zero. The highest sigma story is equal to one. 23 sigma levels are selected for the vertical grid structure with the model top pressure of 100 mb at approximately 15 km. The height of the first 12 levels extends up to 2 km from the surface with the lowest level at approximately 40 m.

### 2.3. Simulation Design and Scenarios

Models-3/CMAQ is a three-dimensional Eulerian atmospheric chemistry and transport modeling system, which can simulate almost all major components including SO_2_, NO_2_, CO, O_3_, PM_2.5_, and PM_10_ throughout the troposphere. The SAPRC-99 chemical mechanism with aqueous and aerosol extensions and AERO5 model are selected for the gaseous chemistry and aerosol modules, respectively. The aqueous-phase chemistry mechanism is the Regional Acid Deposition Model (RADM). It is noted that online dust emissions are not included in CMAQ v4.7.1; that is to say, this paper does not calculate the contribution of dust emission.

In this paper, CMAQ is applied with Brute-Force method as a source sensitivity method for quantifying source contributions of PM_2.5_ by zeroing out emissions from a specific source [18]. This mode will simulate different cases, firstly on base case emission and then on emission of zeroing-out a specific source of region. The discrepancy between the base and the sensitivity simulations can be attributed as the contributions of particular source category. This method is a widely used application way to predict the effect of source [19]. But this method is approximate source contributions [20, 21]. In this paper, base scenarios were preformed firstly using this model, and then zero-out regional source included Beijing-Tianjin, southern Hebei, northern Hebei, Henan, Shanxi, and Shandong for calculating the spatial source contributions of PM_2.5_, respectively. The other scenarios were focused on zeroing the sectoral emission in those regions (i.e., zero-out emissions of industrial source, domestic source, transportation, power plant, and agricultural source of Beijing-Tianjin, resp.). 41 scenarios are simulated to quantify the spatial contributions, the sectoral contributions, and spatial-sectoral contributions in Hebei cities. Additionally, industrial source, domestic source, transportation, power plant, and agricultural source were derived from MEIC emissions inventory, which is a bottom-up emission inventory developed by Tsinghua University [22].

According to the conclusions of BTH-Steel version 1.0 (Emissions Inventory Of Steel Industry in the Beijing-Tianjin-Hebei Area, BTH-Steel version 1.0), the three cities of Xingtai, Handan, and Shijiazhuang are regarded as the SHB in this paper, where the steel and iron industry are centered (http://www.china-eia.com/tzgg/12373.htm). Additionally, the three cities belong to the northern china plain and are located in eastern Taihang Mountain. In this area, the major industrial manufactures include coal, power, steel and iron, and glass industry. Because of the high emission and typical terrain, this area experiences the most severe haze event in January 2013. The other cities (Zhangjiakou, Chengde, Qinhuangdao, Cangzhou, Hengshui, Langfang, Baoding, and Tangshan) in Hebei are seen as the northern Hebei (NHB). Among these cities, most of the northwest areas in Hebei are mountainous and hilly, such as Zhangjiakou, Chengde, and Qinhuangdao. Tangshan and Baoding located around the Beijing and Tianjin city are developing cities. Tangshan is a rapid development city, and the gross domestic product (GDP) of Tangshan in 2013 was up to 612.1 billion RMB and ranked 1st in Hebei, because there are lots of industrial manufactures based on coal and steel, which emit large amounts of air pollutants. There is almost no heavy industry in Cangzhou, Hengshui, and Langfang, but they suffered from the PM_2.5_ pollution.

## 3. Results

### 3.1. The Spatial Contributions of PM_2.5_ Concentrations

This paper used the same model and configurations details introduced by Wang et al. [15]. The model results were thoroughly evaluated in terms of five major meteorological parameters and chemical concentrations (PM_2.5_ and PM_10_). Mean bias (MB), the root mean square error (RMSE), the normalized mean bias (NMB), the normalized mean error (NME), the mean fractional bias (MFB), and the mean fractional error (MFE) were analyzed according to the standard defined in Zhang et al. [23]. MM5-CMAQ could reproduce the most polluted episodes in southern Beijing-Tianjin-Hebei, the Yangtze River Delta, and the Sichuan Basin, respectively. But in northeastern and northwestern China, the concentrations of PM_2.5_ and PM_10_ present underprediction due to the spatial allocation of the emissions and the lack of an online dust emission module in this model. The stimulated results overall underpredict PM_2.5_ and PM_10_ concentrations over Domain 1. Although the MFBs and MFEs to PM_2.5_ reached −19.5% and 58.5% in January and −14.1% and 62.1% in February, respectively, all of them were within the criteria for a satisfactory performance. As for PM_10_, the NMBs were, respectively, −32.0% in January and −31.6% in February. All of the prediction meteorological field, the spatial allocation emissions, and dust emissions might lead to underprediction of PM_2.5_ and PM_10_. In Domain 2, the model predictions agreed well with observations. The averaged NMBs for PM_2.5_ of Beijing, Tianjin, Shijiazhuang, Xingtai, and Handan were −8.2%, 40.6%, 0.8%, −18.4%, and 8.8% in January and 9.4%, 27.3%, −19.5%, −13.9%, and −5.6% in February, respectively. The domain-wide NMBs for PM_10_ over Domain 2 were −13.6% and −10.4% for January and February. The finer grid resolution notably reduced the underpredictions in PM_10_.

**Figure 2:**
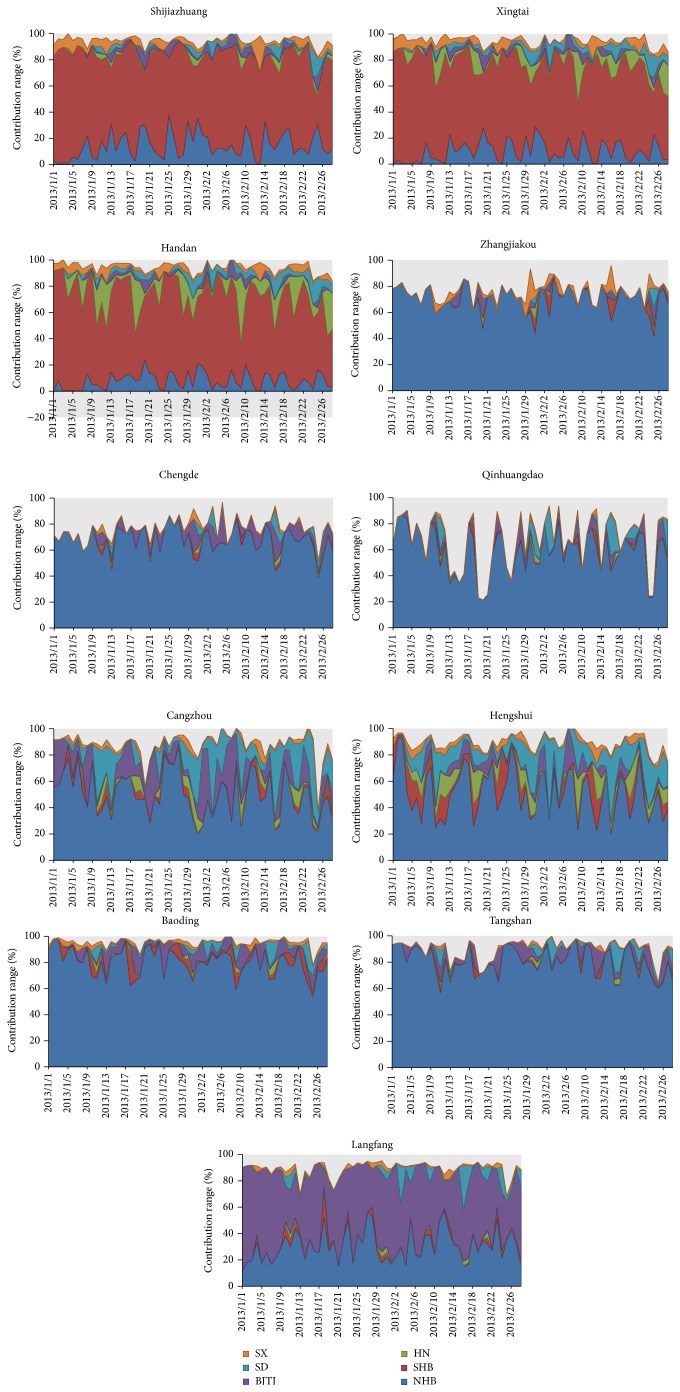
The source contributions ranges by spatial source to PM_2.5_ concentrations in 11 cities of Hebei (SHB: southern Hebei; NHB: northern Hebei; BJTJ: Beijing and Tianjin; SX: Shanxi; SD: Shandong; HN: Henan).

Table 2 gives the average spatial contributions to PM_2.5_ concentrations in Hebei cities during January and February 2013. In Xingtai, Shijiazhuang, and Handan, PM_2.5_ was mainly originated from the SHB with the fractions of 70.6%, 70.8%, and 68.5% in January and 63.9%, 66.4%, and 63.0% in February, respectively. That is to say, the local emission is the major contributor. In Shijiazhuang, NHB contributed 14.1% and 15.0% of PM_2.5_ concentrations in January and February, which were higher than 9.4% and 10.1% in Xingtai and 7.6% and 7.9% in Handan. The source contributions of Henan (HN) to PM_2.5_ in January and February were, respectively, 5.6% and 7.6% in Xingtai and 9.9% and 10.9% in Handan, both of which were higher than 1.4% and 1.9% in Shijiazhuang. Geography position plays an important role. Therefore, HN contributed more PM_2.5_ in Handan, and NHB contributed more PM_2.5_ in Shijiazhuang shown in Figure 2. This paper noted that HN is the largest contributor before and after January 11 identified as the heaviest day, but SHB is the major contributor on January 11. PM_2.5_ concentration would decrease shortly when the contributions of HN increase in Handan. Additionally, the contribution of NHB would not develop a high PM_2.5_ concentration despite the fact that its contribution is not ignored. High PM_2.5_ concentrations usually occurred under high contribution of SHB. Namely, local emission should be firstly controlled.

In Zhangjiakou, Chengde, and Qinhuangdao, NHB was the largest contributor shown in Figure 2, which contributed 69.8% and 70.7% on average to Zhangjiakou in January and February, 68.7% and 66.2% to Chengde, and 57.7% and 59.6% to Qinhuangdao, respectively. Little difference of contributions between the two months suggests that there is no intensive emission and no notable regional transport occurred in January. Stagnant meteorological conditions, such as low wind speed and inversion layer, resulted in accumulation of particulate matters, as well as high relative humidity that accelerates the reaction of heterogeneous chemistry [24]. Simultaneously, the sum source contributions of January and February were, respectively, 75.1% and 78.2% in Zhangjiakou, 64.4% and 71.7% in Qinhuangdao, and 75.5% and 78.2% in Chengde. Dust emission could not be calculated in this model result in underestimation of source contributions. This paper noted that SD contributed more PM_2.5_ in February with contribution of 5.7% that is higher than 2.0% of January in Qinhuangdao. The upper air of Bohai Sea could produce PM_2.5_ and supply sea-slat into atmosphere, which arrive at Qinhuangdao city along with the particles from SD under southern wind.

In Cangzhou, Hengshui, and Langfang, all of SD, BJTJ, SHB, SX, and HN contributed a considerable amount of PM_2.5_ shown in Figure 2. In Hengshui, NHB contributed 54.3% and 48.6% of PM_2.5_ in January and February. SD was the second largest contributor in January, with contributions of 11.0% to PM_2.5_, followed by 10.2% of SHB and 7.0% of HN. In Langfang, BJTJ was the most obvious contributor that contributed 52.9% and 48.9% of PM_2.5_ in January and February, respectively, which have exceeded 30.0% and 31.5% of NHB. Therefore, the source contributions of the transport processing of BJTJ should be taken into account in making control strategies. In the three cities, the influence of complicated multiple sources induces the high PM_2.5_ in January; that is to say, regional transport process is the major cause for formation of haze event. Therefore, control strategies should be focused on regional joint policy making in the three cities.

In Tangshan and Baoding, NHB contributed 81.6% of January and 79.0% of February in Tangshan and 82.1% and 78.2% in Baoding, respectively. Additionally, the sum contributions were 89.4–91.8% in Tangshan and 95.3-95.4% in Baoding. It is indicated that MM5-CMAQ is available to calculate source contribution to PM_2.5_ in the two cities. And local emissions dominate the source contribution of PM_2.5_. BJTJ is the second largest contributor. This paper inferred that transport process may be the process of northeast and southwest, both of which influence BJTJ and vice versa. Further, Wang et al. [1] suggested that there is transport path of PM_2.5_ from the southern to northern of Hebei, according to analysis of the observed PM_2.5_ concentrations and the results of HYSPLIT4 model. Particulate matters would arrive at Baoding city before it arrived at Beijing city [25, 26]. However, SHB contributed 3.7% of PM_2.5_ in January and 3.5% of PM_2.5_ in February. Therefore, more attention should be paid to local emission reduction.

### 3.2. The Sectoral Contributions to PM_2.5_ Concentrations

Table 3 gives the average sectoral contributions to PM_2.5_ concentrations in Hebei cities; the result showed that domestic, industrial, and agricultural emissions are the top three sources in SHB from January to February 2013; and the contributions of power plants and transportation were relatively small. Such as in Handan, domestic emissions contributed 40.7% of PM_2.5_ in January and decreased to 35.1% in February. Yet industrial and agricultural emissions contributed 35.4% and 17.4% of PM_2.5_ in January, which slightly increased to 37.0% and 20.9% in February. Similar variations are found in Xingtai and Shijiazhuang. The reason is that coal combustion and intensive industries emit amounts of particulate matters in the three cities. On the other hand, typical terrain and position, weekly meteorological conditions are not conducive to diffusion of particulate matters. All of them promote the development and formation of the heaviest haze episode.

**Figure 3:**
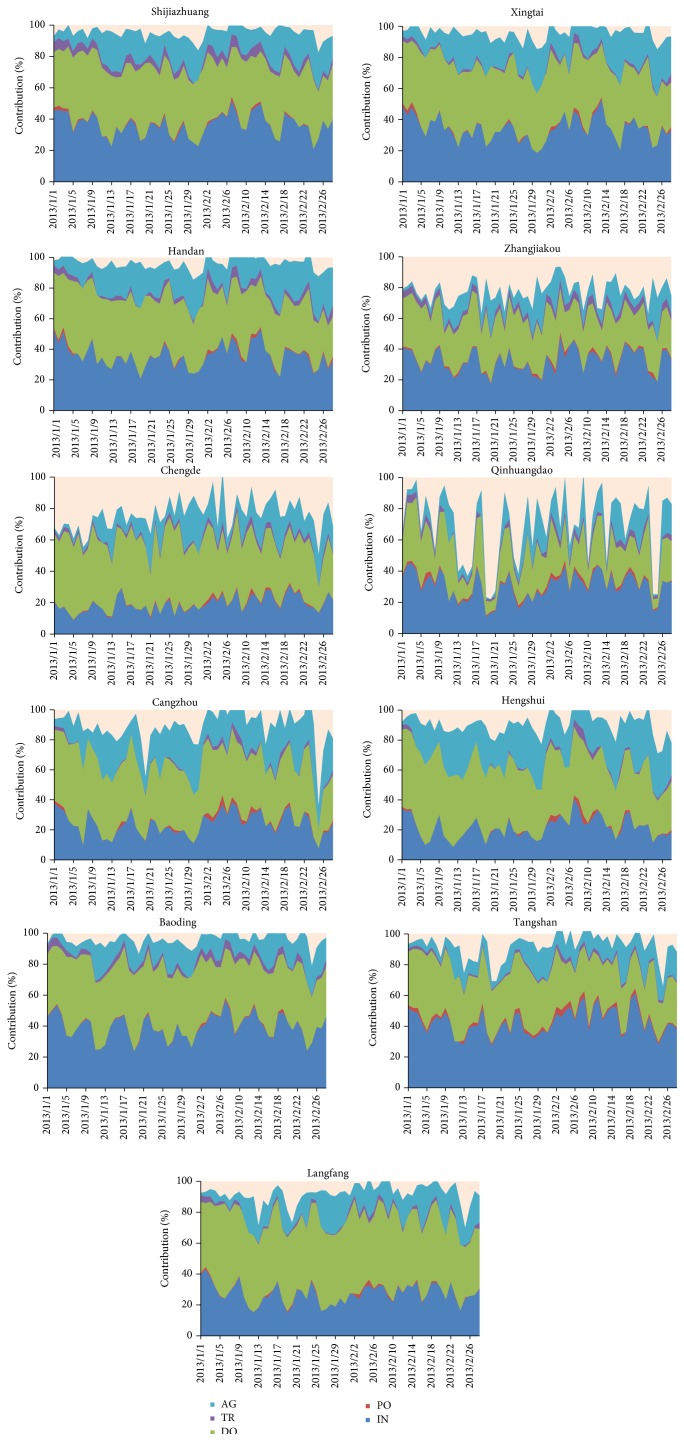
The contribution of sectors to PM_2.5_ concentrations in Hebei from 1 January to 28 February 2013.

Domestic, industrial, and agricultural emissions are the top three contributors in Zhangjiakou, Chengde, and Qinhuangdao as shown in Figure 3. But the contributions of industrial and agricultural emissions are relatively low, compared to the SHB cities. The source contributions of industrial emissions are 16.7% and 21.9% of PM_2.5_ in January and February in Chengde, which are lower than in Zhangjiakou and Qinhuangdao. It is related to economic structure in Chengde where tourism is always pillar industry. As for domestic emissions, the source contribution in Zhangjiakou (31.3%) and Qinhuangdao (25.3%) is significantly lower than 43.1% of PM_2.5_ in January in Chengde. Thus, more attention should be paid to domestic emissions in Chengde.

In Hengshui, the source contributions of domestic emissions were, respectively, 46.5% and 39.0% to PM_2.5_ in January and February, followed by 23.7% and 26.7% of agricultural emissions and 21.3% and 24.4% of industrial emissions. It is worthy of concern that the contributions of agricultural emissions exceed industrial emissions and have been the secondary largest contributor results from fewer industries located in Hengshui. Regional transport process is an important impact factor; that is the neighboring SD province is the largest agricultural province, which would bring amounts of PM_2.5_ to Hengshui. Additionally, agricultural emissions are important sources of ammonia, which react into ammonium in pattern of fine particulate matter in the atmosphere. Therefore, the control of agricultural emissions should be considered in here. In Cangzhou, domestic emissions contributed 43.6% and 38.0% of PM_2.5_ concentrations in January and February, respectively, followed by 22.7% and 26.7% of industrial emissions and 20.8% and 24.3% of agricultural emissions. Although the contributions of agricultural emissions ranked number three, its contributions were slightly lower than industrial emissions. Similar to Cangzhou and Hengshui, domestic emissions were the largest contributor, which contributed 49.0% and 47.0% in January and February in Langfang, respectively, followed by 26.6% and 28.6% of industrial emissions. But agricultural emissions contributed 12.9% and 16.1% of PM_2.5_, which were lower than in Cangzhou and Hengshui. In summary, domestic emissions dominate the contributions of PM_2.5_ in the three cities, especially for Langfang. At the same time, this paper found that more attention should be paid to agricultural emissions, compared to other cities.

Tangshan and Baoding are different from the other cities of Hebei; industrial emissions were the largest contributor and contributed 39.8% and 45.8% of PM_2.5_ in January and February in Tangshan and 38.1% and 41.9% in Baoding, respectively. As statistic of China Environmental Impact Assessment (http://www.china-eia.com/tzgg/12373.htm), emission inventory of steel presents that a number of industry are located in Tangshan and Baoding, especially for Tangshan, considering that steel and coal industry are pillar industry in Tangshan and Baoding. Domestic emission contributed 36.4% in January and 30.2% in February to Tangshan and 42.0% and 36.9% to Baoding. Therefore, industrial and domestic emissions should be controlled in Tangshan and Baoding, especially for industrial emissions.

### 3.3. The Spatial-Sectoral Contributions to PM_2.5_ Concentrations

Industrial emissions of SHB contributed 29.7%, 27.3%, and 28.8% of PM_2.5_ to Shijiazhuang, Xingtai, and Handan. Domestic emissions of SHB contributed 24.6%, 27.6%, and 25.1% of PM_2.5_ to Shijiazhuang, Xingtai, and Handan, respectively. In addition to the source contribution of SHB, domestic emissions of NHB contributed nonignorable PM_2.5_ with 8.0%, 5.1%, and 4.1% to Shijiazhuang, Xingtai, and Handan. The great importance of establishing a regional joint framework of policy making and action system would mitigate air pollution in this area, but control of local emission should be considered firstly.

In Zhangjiakou and Qinhuangdao, the largest contributor was industrial emissions of NHB, with contributions of 31.2% and 28.2%, followed by 26.9% and 20.1% of domestic emissions of NHB and 8.5% and 8.2% of agricultural emissions of NHB as shown in Table 4. As for Chengde, domestic emissions of NHB contributed 35.8% to PM_2.5_ concentrations, which was obviously higher than 16.9% of industrial emissions of NHB and 10.8% of agricultural emissions of NHB. Chengde was easily influenced by BJTJ, compared to Zhangjiakou and Qinhuangdao. Therefore, domestic emissions of BJTJ contributed 2.8% of PM_2.5_ to Chengde, which were higher than in Zhangjiakou and Qinhuangdao. That is, there may be amounts of hills in Zhangjiakou and Qinhuangdao, which affect the transport process of PM_2.5_ and decrease thus the source contribution of BJTJ to them.

The contribution of multiple sectors is an obvious characteristic in Cangzhou, Hengshui, and Langfang. This paper noted that 7.5% of domestic emissions of BJTJ, 5.8% of domestic emissions of SD, 5.3% of industrial emissions of BJTJ, and 4.6% of agricultural emissions of SD contributed to Cangzhou. More detailed contributions were presented in Table 4. Hengshui was similar to Cangzhou; domestic emissions of NHB contributed 24.6% of PM_2.5_ concentrations, followed by 13.5% of industrial emissions of NHB, 11.1% of agricultural emissions of NHB, 6.9% of domestic emissions of SD, 4.7% of domestic emissions of SHB, and 4.6% of agricultural emissions of SD. In comparison with Hengshui, Cangzhou was easily influenced by BJTJ and SD, but Hengshui was more easily influenced by SHB and SD. As for Langfang, 29.4% of domestic emissions of BJTJ were the largest contributor, followed by 16.0% of industrial emissions of BJTJ, 14.9% of domestic emissions of NHB, and 9.1% of industrial emissions of NHB. Similar to Cangzhou and Hengshui, regional joint policy making should be implemented in Langfang, especially for concerning the contributions of BJTJ. Thus, controls of emissions in the neighborhood regions should be further meaningful for mitigating PM_2.5_ pollution in Cangzhou, Hengshui, and Langfang.

As for Baoding and Tangshan, industrial emissions of NHB were the most essential contributor, with contributions of 35.4% and 40.0%, followed by 31.9% and 28.6% of domestic emissions of NHB and 9.2% of agricultural emissions of NHB, respectively. Controls of industrial emissions of NHB should be considered firstly, especially for Tangshan.

## 4. Conclusion

This paper used MM5-CMAQ model to assess the source contributions of PM_2.5_ in Hebei cities. In southern cities of Hebei, SHB contributed 70.6%, 70.8%, and 68.5% of PM_2.5_ to Xingtai, Shijiazhuang, and Handan in January, respectively. Because of the geography situation, NHB contributed more PM_2.5_ to Shijiazhuang, and HN contributed more PM_2.5_ to Handan and Xingtai. In the three cities, domestic emissions were the most important contributors in January, with contributions of 39.4% to Shijiazhuang, 42.4% to Xingtai, and 40.7% to Handan. Simultaneously, industrial and agricultural emissions were nonignored sources. As for the contributions of regions and sectors, the local emissions are major sources.

In Zhangjiakou, Chengde, and Qinhuangdao, NHB was the most essential spatial contributors, which contributed 69.8%, 68.7%, and 57.7% of PM_2.5_ in January, respectively. And domestic and industrial emissions were the major sectoral contributors. Domestic emissions contributed 31.3% and 27.3% of PM_2.5_ to Zhangjiakou, 43.1% and 36.4% to Chengde, and 25.3% and 22.9% to Qinhuangdao in January and February, respectively. Furthermore, domestic and industrial emissions of NHB are the most important spatial-sector sources.

NHB was the most significant contributor in Cangzhou, with the fractions of 54.1% and 45.9% of PM_2.5_ in January and February. This paper found that BJTJ and SD contributed 14.0% and 10.7% of PM_2.5_ to Cangzhou in January, which cannot be ignored. Similar to Cangzhou, the outside regions contributed quite a part of PM_2.5_. It was found that more complicated multiple sources contributions induce the high PM_2.5_ in the three cities. That was to say, regional transport process is the major cause for formation of haze event. Therefore, control strategies should be focused on regional joint policy making in here. Additionally, more attention should be paid to agricultural emissions in Cangzhou and Hengshui, which contributed 20.8% and 24.3% to Cangzhou and 23.7% and 26.7% of PM_2.5_ to Hengshui in January and February, respectively.

In Baoding and Tangshan, the most essential contributors of NHB contributed 82.1% and 78.2% of PM_2.5_ to Baoding and 81.6% and 79.0% to Tangshan in January and February. Domestic and industrial emissions were the largest contributors in Baoding (42.0% and 38.1% in January) and Tangshan (36.4% and 39.8% in January). Industrial and domestic emissions of NHB were the major contributors, with contributions of 35.4% and 31.9% of PM_2.5_ to Baoding and 40.0% and 28.6% of PM_2.5_ to Tangshan. All in all, industrial and domestic emissions should be considered in Tangshan and Baoding, especially for industrial emissions controls of emissions of NHB.

## Figures and Tables

**Table 1:** The source contribution (%) of PM_2.5_ in different cities of China.

	Regional	Local				
		Coal combustion	Industry	Dust	Transportation	Others
Beijing	28–36	22.4	18.1	14.3	31.1	14.1
Tianjin	22–34	27	17	30	20	6
Shijiazhuang	23–30	28.5	25.2	22.5	15.0	8.8
Jinan	20–32	27	18	24	15	16
Shanghai	16–36	13.5	28.9	13.4	29.2	15.0

Beijing: http://www.zhb.gov.cn/zhxx/hjyw/201404/t20140416_270592.htm.

Tianjin: http://www.tjhb.gov.cn/news/news_headtitle/201410/t20141009_570.html.

Shijiazhuang: http://www.cnemc.cn/publish/totalWebSite/news/news_42659.html.

Jinan: http://www.cnemc.cn/publish/totalWebSite/news/news_43773.html.

Shanghai: http://www.cenews.com.cn/sylm/hjyw/201501/t20150109_786239.htm.

**Table 2:** The average spatial source contribution (%) to PM_2.5_ concentrations in Hebei cities.

		SHB	NHB	BJTJ	SX	SD	HN	Sum
Shijiazhuang	Jan.	70.8	14.1	2.3	4.9	1.4	1.4	94.9
	Feb.	66.4	15.0	3.2	3.8	3.8	1.9	94.1

Xingtai	Jan.	70.6	9.4	2.2	4.9	2.5	5.6	95.2
	Feb.	63.9	10.1	3.2	3.6	5.8	7.6	94.2

Handan	Jan.	68.5	7.6	1.9	4.1	2.9	9.9	94.9
	Feb.	63.0	7.9	2.8	2.9	6.2	10.9	93.7

Zhangjiakou	Jan.	0.5	69.8	1.6	2.5	0.2	0.5	75.1
	Feb.	2.0	70.7	1.6	2.7	0.8	0.4	78.2

Chengde	Jan.	0.7	68.7	4.0	1.1	0.6	0.4	75.5
	Feb.	0.7	66.2	7.6	0.7	2.4	0.6	78.2

Qinhuangdao	Jan.	0.8	57.7	2.7	0.6	2.0	0.6	64.4
	Feb.	0.8	59.6	4.4	0.6	5.7	0.6	71.7

Cangzhou	Jan.	3.1	54.1	14.0	2.1	10.7	3.7	87.7
	Feb.	2.6	45.9	16.1	2.0	18.7	3.1	88.4

Hengshui	Jan.	10.2	54.3	4.2	3.0	11.0	7.0	89.7
	Feb.	6.2	48.6	6.5	2.7	18.7	6.3	89

Langfang	Jan.	2.3	30.0	52.9	1.5	1.4	0.8	88.9
	Feb.	1.5	31.5	48.9	1.3	5.9	0.8	89.9

Baoding	Jan.	3.7	82.1	5.5	1.8	1.3	0.9	95.3
	Feb.	3.5	78.2	6.8	1.4	4.4	1.1	95.4

Tangshan	Jan.	0.8	81.6	4.7	0.6	1.2	0.5	89.4
	Feb.	0.9	79.0	6.3	0.6	4.4	0.6	91.8

SHB: southern Hebei; NHB: northern Hebei; BJTJ: Beijing and Tianjin; SX: Shanxi; SD: Shandong; HN: Henan.

**Table 3:** The sectoral contributions to the PM_2.5_ concentrations in 11 cities of Hebei.

		PO	IN	DO	TR	AG
Shijiazhuang	Jan.	0.5	35.1	39.4	4.1	15.4
	Feb.	0.4	38.0	36.3	4.4	17.5

Xingtai	Jan.	0.0	33.6	42.4	1.2	17.3
	Feb.	0.7	35.5	38.1	2.4	20.8

Handan	Jan.	−0.1	35.4	40.7	2.2	17.4
	Feb.	1.0	37.0	35.1	4.0	20.9

Zhangjiakou	Jan.	0.9	30.4	31.3	3.7	9.7
	Feb.	1.4	35.1	27.3	4.6	11.2

Chengde	Jan.	0.4	16.7	43.1	2.0	11.5
	Feb.	1.1	21.9	36.4	3.1	16.5

Qinhuangdao	Jan.	1.1	28.7	25.3	2.6	9.0
	Feb.	1.7	33.0	22.9	3.5	13.5

Cangzhou	Jan.	−0.4	22.7	43.6	0.1	20.8
	Feb.	1.7	26.7	38.0	2.4	24.3

Hengshui	Jan.	−1.8	21.3	46.5	−0.4	23.7
	Feb.	1.2	24.4	39.0	2.1	26.7

Langfang	Jan.	0.0	26.6	49.0	1.4	12.9
	Feb.	0.6	28.6	47.0	2.0	16.1

Baoding	Jan.	−0.1	38.1	42.0	3.0	11.8
	Feb.	0.5	41.9	36.9	3.8	14.8

Tangshan	Jan.	1.8	39.8	36.4	1.4	10.4
	Feb.	2.3	45.8	30.2	2.2	14.4

**Table 4:** The average source contributions (%) to PM_2.5_ concentrations in Hebei cities by source regions and sectors from 1 January to 28 February 2013.

	IN	PO	DO	TR	AG	IN	PO	DO	TR	AG	IN	PO	DO	TR	AG
	Shijiazhuang					Xingtai					Handan				
SHB	29.7	0.6	24.6	4.4	9.4	27.3	0.5	27.6	1.8	9.9	28.8	0.6	25.1	3.0	8.8
NHB	3.3	0.0	8.0	0.0	2.7	2.2	0.0	5.1	0.0	2.2	1.7	−0.1	4.1	0.0	1.8
BJTJ	0.9	0.0	1.5	0.0	0.2	1.0	0.0	1.5	0.0	0.3	0.8	0.0	1.2	0.0	0.2
SD	0.4	−0.1	1.3	−0.1	0.9	0.7	−0.1	2.0	0.0	1.3	0.7	−0.1	2.3	0.0	1.4
HN	0.3	−0.1	0.7	−0.1	0.7	1.5	−0.1	2.4	0.0	2.7	2.5	−0.2	3.7	0.0	4.1
SX	1.5	0.1	1.9	0.0	0.5	1.6	0.1	1.9	0.0	0.5	1.3	0.0	1.6	0.0	0.4
Sum	36.1	0.5	38	4.2	14.4	34.3	0.4	40.5	1.8	16.9	35.8	0.2	38	3	16.7

	Zhangjiakou					Chengde					Qinhuangdao				
SHB	0.2	0.0	0.4	0.0	0.5	0.1	0.0	0.3	0.0	0.3	0.2	0.0	4.2	0.0	0.2
NHB	31.2	0.9	26.9	4.0	8.5	16.9	0.6	35.8	2.4	10.8	28.2	1.3	20.1	3.0	8.2
BJTJ	0.4	0.0	0.8	0.1	0.4	1.5	0.0	2.8	0.1	1.2	1.0	0.0	1.8	0.0	0.5
SD	0.1	0.0	0.2	0.0	0.3	0.2	0.0	0.5	0.0	0.6	0.9	0.0	1.2	0.1	1.4
HN	0.1	0.0	0.1	0.0	0.3	0.1	0.0	0.1	0.0	0.3	0.1	0.0	0.2	0.0	0.3
SX	0.7	0.1	1.1	0.0	0.7	0.3	0.0	0.4	0.0	0.2	0.2	0.0	0.3	0.0	0.1
Sum	32.7	1	29.5	4.1	10.7	19.1	0.6	39.9	2.5	13.4	30.6	1.3	27.8	3.1	10.7

	Cangzhou					Hengshui					Langfang				
SHB	0.6	−0.1	1.5	−0.1	0.8	2.0	−0.2	4.7	−0.1	1.6	0.5	0.0	1.0	0.0	0.5
NHB	14.0	0.3	23.9	0.8	10.4	13.5	0.2	24.6	0.9	11.1	9.1	0.2	14.9	0.3	5.0
BJTJ	5.3	0.2	7.5	0.3	1.1	1.9	0.0	2.7	0.0	0.4	16.0	0.2	29.4	1.4	3.6
SD	2.9	0.2	5.8	0.1	4.6	2.6	−0.2	6.9	−0.1	4.6	0.7	0.0	1.5	0.0	1.1
HN	0.5	−0.2	1.3	−0.1	1.6	0.2	−0.3	2.8	−0.2	2.8	0.1	−0.1	0.3	0.0	0.4
SX	0.7	0.0	1.0	0.0	0.3	1.0	−0.1	1.5	0.0	0.4	0.4	0.0	0.7	0.0	0.2
Sum	24	0.4	41	1	18.8	21.2	−0.6	43.2	0.5	20.9	26.8	0.3	47.8	1.7	10.8

	Baoding					Tangshan									
SHB	0.9	−0.1	1.8	−0.1	0.8	0.2	0.0	0.4	0.0	0.2					
NHB	35.4	0.3	31.9	3.4	9.2	40.0	2.0	28.6	1.7	9.2					
BJTJ	2.2	0.0	3.3	0.0	0.4	1.5	0.0	3.0	0.0	0.8					
SD	0.5	−0.1	1.3	0.0	0.9	0.5	0.1	1.0	0.0	1.0					
HN	0.2	−0.1	0.4	0.0	0.5	0.1	0.0	0.2	0.0	0.3					
SX	0.5	0.0	0.7	0.0	0.2	0.2	0.0	0.3	0.0	0.1					
Sum	39.7	0.0	39.4	3.3	12	42.5	2.1	33.5	1.7	11.6					
